# Tele-psychotherapy during the COVID-19 pandemic: a mini-review

**DOI:** 10.3389/fpsyt.2023.1060961

**Published:** 2023-07-05

**Authors:** Nicolas Tajan, Maud Devès, Rémy Potier

**Affiliations:** ^1^Laboratory of Psychopathology and Psychoanalysis, Graduate School of Human and Environmental Studies, Kyoto University, Kyoto, Japan; ^2^Institut de Physique du Globe de Paris, CNRS, Université Paris Cité, Paris, France; ^3^Centre de Recherche Psychanalyse Médecine et Société, CNRS, Université Paris Cité, Paris, France; ^4^Centre de Recherche en Psychopathologie et Psychologie Clinique, Institut de Psychologie, Université Lumière Lyon 2, Lyon, France

**Keywords:** telemedicine, psychotherapy, mental health services, PRISMA, digital mental health

## Abstract

The COVID-19 pandemic has dramatically changed psychotherapy practices. Psychotherapy around the world has shifted from predominantly face-to-face settings to overwhelmingly online settings since the beginning of the pandemic. Many studies have been published on this topic, but there has been no review of the literature focused on the experience of psychotherapists. Our goal was to identify the challenging issues of teletherapy, including the efficiency of online consultations and the extent to which they are accepted by therapists and patients. A PubMed literature search using the [(“Teletherapy” OR “Telebehavioral health” OR “telepsychotherapy”) AND (“COVID-19”)] search string retrieved 46 studies focused on mental health professionals, as detailed in a PRISMA flow diagram. Two reviewers independently screened the abstracts and excluded those that were outside the scope of the review. The selection of articles kept for review was discussed by all three authors. Overall, the review contributes to the description and evaluation of tele mental health services, including teletherapy, online counseling, digital mental health tools, and remote monitoring.

## 1. Introduction

The COVID-19 pandemic has dramatically changed psychotherapy practices. Psychotherapy around the world has shifted from predominantly face-to-face settings to overwhelmingly online settings since the beginning of the pandemic. Many initiatives worldwide have been implemented targeting healthcare workers in response to the COVID-19 pandemic, such as psychological support systems and hotlines for hospital workers (1). While there is an urge to provide emotional support to the healthcare workforce (2) and protect their mental health (3) there have been no studies specifically investigating mental health care workers’ own mental health. This paper aims to explore the consequences of the shift to telehealth in mental health practice for both clinicians and their patients.

The COVID-19 pandemic has been viewed as an opportunity to transform psychiatric care through the implementation of telehealth (4). However, there are several challenging issues regarding the implications of telehealth, including digital health inequity (5), and we do not have sufficient knowledge on the efficiency of online consultations and the extent to which they are accepted by clinicians and patients (6). More generally, the issue of distance mental health care practice is also central to disaster research (7, 8). Supporting people affected by health, but also natural, man-made and technological disasters, often requires creativity, as traditional care infrastructures and private mental health care practitioners’ offices can be damaged; and, most people today, including many of the most disadvantaged, have access to a phone or the internet (9, 10).

Many studies have been published since the beginning of the pandemic, but there has been no review of the literature focused on mental health providers. Our goal is to identify the challenging issues surrounding teletherapy, including the efficiency of online consultations and the extent to which they are accepted by both therapists and patients.

## 2. Methods

**Figure 1:**
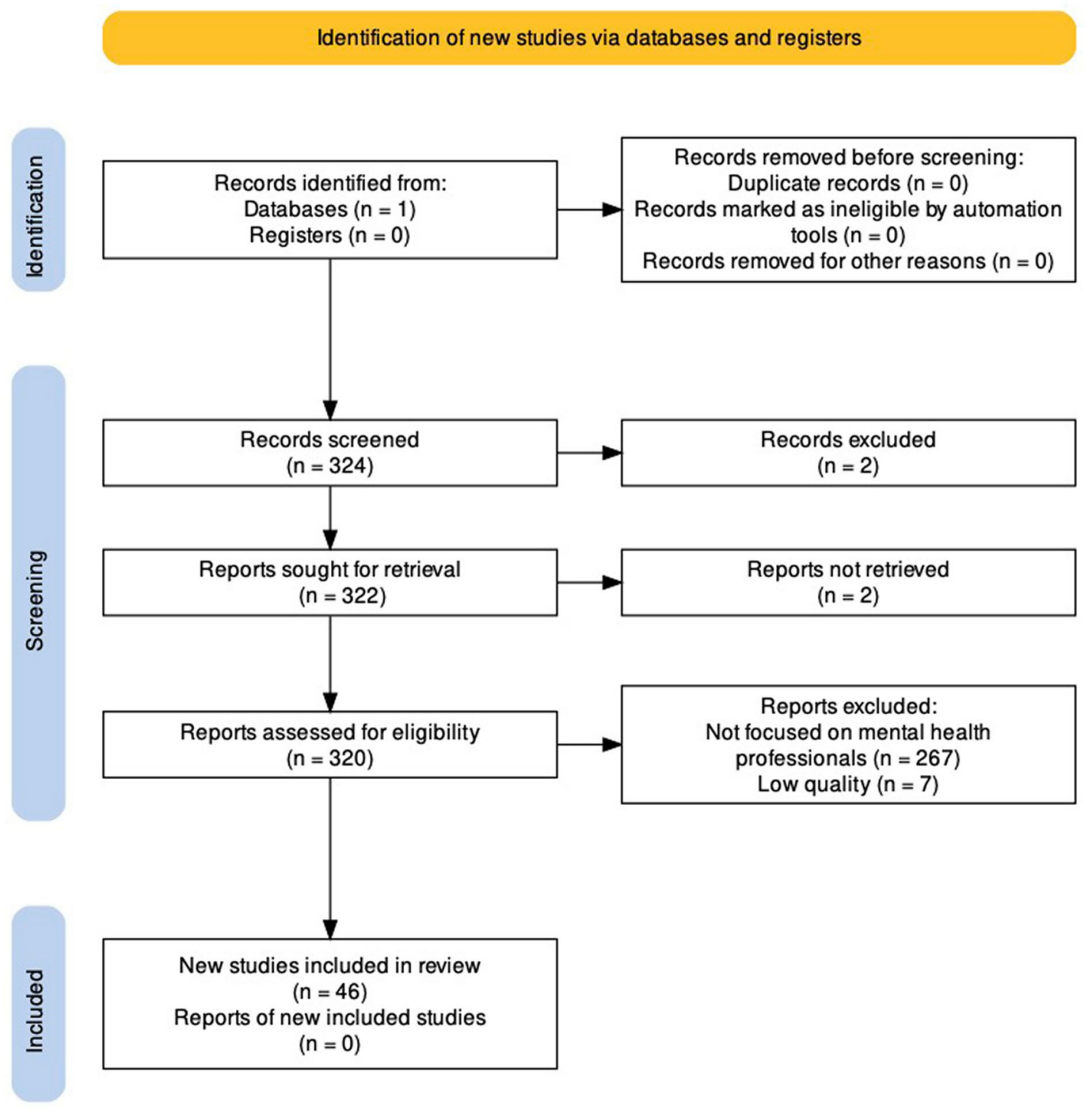
PRISMA 2020 flow diagram. Adapted from Haddaway et al. (11).

A PubMed literature search restricted to the English language using the [(“Teletherapy” OR “Telebehavioral health” OR “Telepsychotherapy”) AND (“COVID-19”)] search string retrieved 46 studies focused on mental health professionals, as detailed in a PRISMA flow diagram (11). The articles under review were published from January 1, 2020 to April 13, 2023 (Figure 1).

Two reviewers (NT and RP) independently screened the abstracts and excluded those that were outside the scope of the review. To be included in the mini-review, articles had to be focused on mental health professionals and online psychotherapy practice during the Covid-19 pandemic. Articles targeting other health care workers or providers, patients or their parents or caregivers were excluded. The selection of articles selected for review was discussed by all three authors via Zoom (Table 1).

**Table 1:** Synthesis of the studies included in the Mini-Review.

	Authors	Country	Number of participants	Profession of participants	Methods	Focus	Challenging issues
Mental health services	Feijt, M. et al.	Netherlands	51	Mental health care professionals	Online qualitative survey of 3 closed questions and 5 open questions	Mental health care	Insufficient technological infrastructure (internet-connection stability, availability of smartphone or computer), difficulty with verbal and nonverbal communication, lack of privacy
	Pugh, et al.	N/A	41	Experts in CBT, CFT, EFT, psychodrama, schema therapy, and voice dialog	Brief questionnaire: email survey of 4 open ended questions	Guidelines for facilitating tele-chairwork	Incorporation of “chairwork,” in teletherapy
	Probst, et al.	Austria	1547	Licensed psychotherapists	Online survey	Stress and job-related anxiety	Preventing psychotherapists’ burnout
	Lin, T., et al.	USA (+Canada, Germany, Italy Switzerland)	440	Therapists and trainees	Exploratory factor analysis (EFA) confirmatory factor analysis (CFA)	Therapeutic skills	Being forced in teletherapy, opportunities of training in teletherapy, confidence of psychotherapists
	Roberts, et al.	Canada	8	Fly-in and fly-out (FIFO) mental health service providers	Participatory action research, semi-structured interviews, thematic analysis	Inuit Nunangat area, service providers	Lack of technology, internet access, privacy
	Messler et al.	USA	326	Clinical neuropsychologists	Online survey	TeleNP acceptance 3 years after the pandemic	Although teleNP where the patient is located in clinic is feasible and acceptable it is not the case for forensic neuropsychology practice
	Hewitt et al.	USA	16	Neuropsychologists	Commentary	Neuropsychologists’ experience	Healthcare disparities
	von Wirth et al.	Germany	228	Parents and psychotherapists	Follow up survey	Satisfaction with videoconference-delivered CBT for children and adolescents with MD	Children with attentional difficulties, technical difficulties, difficulty maintaining a positive therapeutic relationship
	Winter et al.	Austria	1547, 238, 510	Psychotherapists	3 cross sectional online surveys	Assess patient numbers	Increase workload of psychotherapists and insufficient support of psychotherapists
	Baker et al.	USA	250	Psychotherapists	Online survey	Traumatized children	Screen fatigue, attention span of children, insufficient training in teletherapy
	Khanna et al.	USA	70	Providers working with Veterans	Semi structured interviews, content analysis, rapid approach	Veteran’s self-care skills	Need to increase access of telehealth for all
	Griffith et al.	Scotland	20	Mental Health workers	Online semi-structured interviews, inductive thematic analysis	Mental health workers’ experience of delivering service remotely	Confidentiality cannot be assured for MH workers and patients; MH workers are less able to receive support from colleagues; work-life boundaries are blurred
	Al-Mahrouqi et al.	Oman	6	Clinical psychologists	Semi-structured interviews	Experience using tele- mental health	Lack of public tele-mental health services and guidelines, shortage of trained therapists, limited access to high-speed Internet and electronic devices, privacy and security concerns
	Vitiello and Sowa	USA	111	Clinicians in ambulatory care (Psychiatry department)	Survey	Tele-behavioral Health safety planning	Issues around civil commitment for clinicians who are not familiar with telehealth practice
	Tohme et al.	Lebanon	73	Mental Health professionnals	Online survey	Predictors of use and perceived level of comfort using teletherapy	Lack of awareness and training
	Mishkin et al.	USA	333	Consultation-Liaison psychiatrists, physicians, and others	Online survey, multivariable regression modeling	Adaptation to remote care	Vulnerable populations: sexual and gender minorities
	Boldrini et al.	Italy	306	Licensed psychotherapists	Online survey	Rate of interrupted treatment, satisfaction with teletherapy	CB therapists report higher interruption rate; access a private space at home, use video-conferencing instead of telephone
Psychodynamic psychotherapy	Ronen-Setter, et al.	N/A	2	Psychodynamic psychotherapists	Theoretical article	Accelerated Experiential Dynamic Psychotherapy	Taking more time with patients starting therapy online to discuss the rules regarding the setting, stability, privacy
	Svenson	N/A	1	Psychoanalysts	Essay	Countertransference	Difficulty in relating to the other, loss of control of the frame, personal transferences to technology, fear of personal constrictions on their bodies
	Wang, et al.	USA and China	329	China American Psychoanalytic Alliance (CAPA) and US practitioners	Online survey	Expert opinions about teletherapy	Low familiarity with videoconferencing
	Levey	N/A	1	Psychoanalysts	Essay	Tele-analysis	Complicating notions about distance and intimacy, affective coregulation in intersubjective relating
	Gordon, et al.	56 countries	1490	Psychodynamic practitioners	Survey	Psychodynamic therapy	N/A
	König et al.	Argentina	9	Psychotherapists	qualitative study in-depth interviews, content analysis	Therapy setting, therapeutic relationship, burden	Telepsychotherapy is more exhausting than in-person psychotherapy, need for improvement of technical conditions and privacy during sessions
	Békés et al.	USA	31	Psychotherapists	Semi structured interviews, consensual qualitative research method	Therapists’ subjective experience, therapeutic relationship and process	More active, informal, relaxed and directive in sessions, while missing the energy and intimacy of in-person sessions; Process-level research and subsequent training is needed
	Tyminski	N/A	1	Psychotherapists, psychoanalyst	Essay	Teletherapy with children, adolescents	« Containing » (Bion, Ogden) is challenging online due to the lack of embodied presence
	Schen et al.	N/A	3	Psychotherapists (psychodynamic)	Essay	Psychodynamic psychotherapy (in person, remote and hybrid)	N/A
	Aafjes van Doorn et al.	N/A	1450	Psychodynamic and psychoanalytically oriented therapists	Survey	Transition to online therapy during the first weeks of the pandemic and two months later	Wide range of relational and technical challenges, more tired, less confident and competent, and less connected and authentic, distraction in sessions which increased over time. Younger therapists reported more challenges in the transition to online therapy
	Mancinelli et al.	Italy	281	Licensed psychotherapists	Online survey	Psychotherapists’ self-perception	Fatigue, greater conversational and directive attitude during sessions
Couple marriage and family therapy	Hertlein, et al.	USA, UK, Australia Canada	N/A	Couple/marriage and family therapists	Essay	Couple/marriage and family therapy, recommendations, TBH competencies	Legal and regulatory issues, reimbursement of tele-therapy
	Hardy et al.	USA	58	Couple therapists	Mixed methods	Advantages, challenges, recommendations for practice, implications for clinical training in couple therapy	Couples with violence, severe MDs, suicidal ideation, trauma, substance abuse, client discomfort, clinician fatigue, ethical dilemmas, safety, privacy, emergencies
	Morgan, et al.	USA	2	University training programs in marriage and family therapy	Chi-square analyses, t-test, logistic regression and multiple linear regression model	Transition to teletherapy	Trainees’ struggle to adjust, work with clients who reject teletherapy (confidentiality or privacy issues), or feel the loss of an in-person connection
	McKee, et al.	USA	626	Family systems therapists	Online study	Teletherapy practice and training	Physical medicine and rehabilitation, antisocial personality disorder, traumatic brain injury, family conflict
	Mc Kenny, et al.	UK	312	Systemic therapists	Mixed methods, online survey	Teletherapy and systemic therapy	Digital exclusion, fatigue, isolation, patients’ lack of motivation, disruptions in the flow of therapy, use of therapeutic techniques or resources
	Bate, et al.	N/A	2	Psychotherapists	Theoretical essay	Mentalization-Based Treatment for Children	Children’s mentalization
	Eppler	N/A	55	Clinicians	Reflexive thematic analysis	Systemic therapy	Therapists’ discomfort: eyestrain, blurred vision, motion sickness, emotional vulnerability, fatigue, low energy
	Robbins et al.	USA	N/A	Family therapists	Implementation framework	Functional Family therapy, service delivery	Reimbursement rates, local or state regulations, referral issues; accessing external resources; discharging families; shifting training, consultation, and clinical practice guidelines; experiential, relationship-building aspects of in-person training are more challenging to replicate in a virtual webinar
	Burgoyne, et al.	N/A	N/A	Child, adolescent, couple and family therapists	Essay	Teletherapy for children, adolescents, families and couples	Eye contact, children with ADHD, couples therapy (cases of domestic violence)
	Grames et al.	USA	N/A	Couple/marriage and family therapists	Essay	Tele-supervision and digital training	Difficult to Identify group dynamics, safety, confidentiality; distractions (e.g., children, partners)
Mental disorders	Taylor, et al.	N/A	N/A	Therapists	Commentary	Recommendations for Telehealth Eating disorders services	Discontinuation in treatment (regulations for colleges to provide distance learning, individuals who move to another state are not able to continue seeing their therapist via telehealth)
	Pires de Oliveira Padilha, et al.	Canada	33	Team leaders of early intervention services (EIS)	Cross-sectional descriptive study design, a 41 questions online survey	First episode psychosis, group interventions	Clinicians’ levels of ease with teletherapy, as well as lack of technical support and availability of telehealth equipment
	Johnsson et al.	Australia	141	Allied health practitioners (speech pathologists, occupational therapists, psychologists, educators, and social workers)	Mixed methods	Autism teletherapy, service delivery	Technical difficulties, internet-connection issues, special-needs children with significant mental health issues and intellectual disabilities
	Smith-MacDonald et al.	Canada	31	Key stakeholders	Descriptive, quantitative, and qualitative thematic analyses.	Trauma therapy	Technological and reimbursement issues, “Zoom fatigue”
	Weiskittle, et al.	USA	21	Clinicians in Geriatric mental health	Survey	Veteran MH needs (e.g., trauma); Group teletherapy intervention	Older patients’ cognitive functioning
	Eguia et al.	Philippines	102	Therapists	Mixed methods	Developmental disorders, Satisfaction with teletherapy and benefits	More time searching for resources online and greater fatigue related to teletherapy
	Choudhury et al.	USA	10	Licensed Mental Health providers	In depth semi-structured virtual interviews, inductive open coding	Adverse Childhood Experiences	Accessing mental health services for ethnic minority and socioeconomically disadvantaged individuals with ACE
	Gangamma et al.	USA	186	Licensed Mental Health professionals	Online survey	Predictors of continued use of teletherapy	Vulnerable groups (lower socioeconomic conditions, Medicaid beneficiaries, and those who seek couple and family therapy). Access to technology, housing, childcare, and need for training for professionals.

## 3. Results

Among the 46 studies retained, four subgroups were distinguished. The first group includes articles on mental health services and therapists (17/46). The second group focus solely on psychodynamic approaches (11/46). The third subgroup consists of studies on couples and marriage and family therapy practices (10/46). The fourth group comprises the treatment of specific mental disorders, such as PTSD or developmental disorders (8/46).

### 3.1. Mental health services: insufficient technological infrastructure, training, privacy, and support of telepsychotherapists

A study in the Netherlands (12) focusing on experiences of psychotherapists, both positive and negative, found that insufficient technological infrastructure both on the side of the therapist (internet-connection stability) and the patient, who might lack necessary devices (smartphone or computer) are significant barriers as well as a lack of privacy in the clients’ environments. The need for an improved technological infrastructure, user-friendly technologies, appropriate devices, stable internet connections and privacy is reported by numerous other studies in different contexts and countries. For instance, Roberts and colleagues (13) investigated the specific situations for healthcare delivery from teams who come from outside the region where services are offered, such as Canadian circumpolar regions. The perceived challenges in the case of services for Indigenous communities were the lack of technology, internet access, and privacy. In Italy, CB therapists (14) also mention difficulties in accessing a private space at home and using videoconferencing instead of telephone. In Oman, a study underlines (15) a lack of public tele-mental health services and guidelines, a shortage of trained therapists – consistent with another survey in Lebanon (16) – as well as limited access to high-speed internet and electronic devices, privacy and security concerns.

Increased stress levels were reported among Austrian psychotherapists compared to a control group during the COVID-19 pandemic (17). Additionally, three Austrian cross sectional online surveys assessing patient numbers show an increasing workload for psychotherapists and an insufficient support of psychotherapists (18). A Scottish study (19) also found that MH workers are less able to receive support from colleagues. In addition to an increased stress and a lack of support, a lack of common therapeutic skills in teletherapy compared to in-person therapy is reported by psychotherapists (20). These skills include the use of various therapeutic techniques, intentional silence, empathy, emotional expression, and conversational tone. Undoubtedly, there is an urgent need for training in teletherapy (21) to improve the confidence of psychotherapists.

In a more positive light, acceptability in neuropsychological practice is good despite some limitations for forensic neuropsychology practice (22), or regarding healthcare disparities (23). Moreover, a study has found potential for improving veteran’s self-care skills but insists on the need to increase access to telehealth for all (24). Importantly, there is a satisfaction with videoconference-delivered CBT for children and adolescents (21). Sessions are shorter and easier to schedule, and team meetings are efficient (12). Surprisingly, older generations were less reluctant to accept teletherapy than expected (20), and techniques that were thought to be reserved for in-person sessions can be incorporated in teletherapy (25).

### 3.2. Positive opinion on psychodynamic teletherapy and teleanalysis

It appears that psychodynamic therapists or psychoanalysts have had a more positive opinion regarding the effectiveness of online sessions since the pandemic began, psychotherapists and psychoanalysts based in China being more positive than those based in the US (26). First-person accounts of psychoanalysts were published (27–29), and clinicians started to use the term “teleanalysis,” comparing the impact of physical distance on psychoanalytic treatment to that of the couch (30, 31). In psychodynamic therapy, whether online or in person, the most important aspects of treatment effectiveness are the same: the therapist’s empathy, warmth, wisdom, and skillfulness and patient’s motivation, insightfulness, and level of functioning (32). Additionally, experts in accelerated experiential dynamic psychotherapy have developed specific techniques to be applied in an online setting, and they insisted on the need to take more time with patients starting therapy online to discuss the rules regarding the setting, stability, and privacy (33). Interestingly, studies have found greater conversational, relaxed and simultaneously more directive attitude during online sessions (31, 34). Limitations are found in an Argentinian study insisting that telepsychotherapy is more exhausting than in-person psychotherapy and that an improvement of technical conditions and privacy during sessions are needed (35).

### 3.3. Benefits and challenges of teletherapy for couples and families

Couples and marriage and family therapists identified benefits for patients living in rural areas and underserved populations, and teletherapy eases the logistics involved in making childcare arrangements, which facilitates access to care for working parents (36). Advantages of teletherapy reported by clinicians included the reduction of expenses, increase in personal time, improved access to meetings, improved accessibility, and flexibility (37). An analysis of existing data collected from two university marriage and family therapy training programs in the US found that most cases converted to teletherapy and that “the number of prior in-person sessions attended significantly predicted conversion to teletherapy” (38). It is consistent with a survey of therapists in an organization that provided more than 35,000 virtual sessions of Functional Family Therapy between March and September 2020 worldwide which found similar rates for treatment completion, number of sessions, and therapist fidelity between 2019 and 2020, suggesting that teletherapy is a viable alternative to in-person session (39). Another study found that in most cases, the shift to online sessions during the pandemic was not a threat to the therapeutic relationship, although patients reported to their therapists that “it felt different.” There was agreement among 80 percent of participants that teletherapy offers good quality of care (40).

While the shift to telepsychotherapy was positive overall, there are barriers to teletherapy with couples such as violence, severe MDs, and suicidal ideation (41). Importantly, couples and family teletherapy are not indicated in the case of family conflict, especially if it implies children in the home would not be supervised during the session (36). There are challenges for certain populations and specific pathologies requiring in-person treatment: “Physical medicine and rehabilitation, antisocial personality disorder, traumatic brain injury, and family conflict were associated with the lowest increases in teletherapy uptake” (36). Couples and marriage and family therapists expressed concerns regarding digital exclusion, fatigue, isolation, lack of motivation for some patients, disruptions in the flow of therapy, and many other difficulties regarding the use of therapeutic techniques or resources (37). More precisely, therapists experienced discomfort, such as eyestrain, blurred vision, and motion sickness, to the extent that several of them doubted their ability to continue providing online sessions full time, given the increase in emotional vulnerability and experiences of fatigue and low energy (42).

The Family Institute at Northwestern University launched its teletherapy services in 2018 and made an important contribution to the field. Notably, they insisted on the preparation of the therapist’s office, which is visible on clients’ screens: “While there may be value to ‘humanizing’ a therapist through what the environment discloses about them personally, some content may be problematic. Such details force a client to interact with the therapist’s personal life in a way that may be activating and, depending on the client and the personal information communicated, may violate boundaries and basic standards for professionalism” (40). A ritual for entering into a therapeutic mindset is recommended on both sides, as well as creating an environment that ensures privacy (e.g., a family member should not enter the room) and reduces distractions (e.g., pets), including using a full-screen browser window during sessions. Eye contact might also be a problem. Even when the therapist looks at the camera, the patient might feel that the therapist’s gaze is directed above or below their face. The biggest challenge encountered was teletherapy with children, where considerable adaptation was required, and difficulty maintaining their attention was a recurrent issue. An efficient strategy with children with ADHD was to provide frequent 5-min breaks. With young children, it is advised that session not exceed 30 min and that the camera show the entire room to allow the child to move around and be seen playing. Teens have shown some creativity in ensuring their privacy, including asking their parents to take a walk during their sessions, having sessions in their parents’ parked car, using a noise generator placed outside of their room, or using headphones, which also contributes to creating rituals for the session. Interestingly, many teens appreciated multiple short sessions rather than only one long session. Couples teletherapy was challenging during the pandemic, especially in cases of domestic violence; many patients wanted to talk, but they were also afraid of the consequences after the end of the virtual session. In couples and family therapy, the online setting necessitates clients speak one at a time, which is a major difference compared to in-person sessions (40).

### 3.4. Mental disorders and telepsychotherapy: significant challenges for special-needs children, first-episode psychosis, older patients, and vulnerable groups

Teletherapy seemed to benefit anxious autism spectrum disorder patients, who found it less invasive, and those who worked considered it easier to integrate into their schedule, though others recognized challenges in conducting therapy this way. There are technical difficulties and internet-connection issues, which do not amount to a limitation to its use, *per se*, but they are a barrier for special-needs children with significant mental health issues and intellectual disabilities (43).

According to a Canadian research, digital health can become a standard delivery mode for trauma therapy (44). Benefits include equity of access to care and stigma reduction; however, technological and reimbursement issues will need to be addressed if a generalized digital health system becomes the new normal. Although US clinicians taking care of veterans also found a limitation regarding considerations for cognitive functioning, especially for older patients (45), teletherapy for veterans is considered feasible, acceptable, effective, and adaptable beyond the pandemic period.

Two studies show difficulties in accessing mental health services for ethnic minority and socioeconomically disadvantaged individuals with Adverse Childhood Experiences (46), and challenges for vulnerable groups (lower socioeconomic conditions, Medicaid beneficiaries, and those who seek couple and family therapy) as well as access to technology, housing, childcare, and need for training for professionals (47).

Significant challenges have been reported with children with attentional difficulties in Germany (48) a result consistent with what is observed in the US (21). Additionally, several systemic therapists offering couples teletherapy in the US mentioned having difficulty working with children online due to their short attention spans (42).

Finally, a survey on early intervention services for first-episode psychosis during the first wave of the COVID-19 pandemic in Quebec found limitations regarding clinicians’ levels of ease with teletherapy, as well as lack of technical support and availability of telehealth equipment (49).

## 4. Discussion

Most of the studies were the first of their kind, given the unprecedented nature of the COVID-19 pandemic and the need for teletherapy to be implemented very rapidly after the start of the pandemic and the related lockdowns. More than ever, studies have insisted that teletherapy is more demanding than face-to-face therapy (35, 44, 50), hence the need for self-care practices for the well-being of therapists (17, 33). Teletherapy has a cost for therapists: mental fatigue, physical symptoms, such as eyestrain, blurred vison, and motion sickness, emotional vulnerability, and isolation (18, 21, 34, 37, 42, 51). The issue of being forced into teletherapy, both on the side of patients and therapists, was discussed (20, 38, 52) and should not be disregarded. Among the barriers, concerns over a lack of confidentiality and privacy were mentioned often by patients who were reluctant to use teletherapy. Additionally, the possibility that a patient could identify the home address of their therapist is an important matter, as this could threaten the safety of teletherapists.

There are difficulties in cases of family conflict, where there is a duty to report child abuse and suicidality in minor patients and geriatric populations. It is also difficult to identify neglect or abuse if someone is only treated through teletherapy, especially in cases of, for example, physically impaired and vulnerable populations (36, 47, 53). Family therapy and therapy for psychotic symptoms, severe anxiety, trauma, or individuals in crisis is less suited to online sessions (12).

This review was conceived as a first overview of an unexplored subject and was not intended to be exhaustive. A systematic review of the literature is necessary with a more varied choice of keywords, allowing for the inclusion of relevant articles that were not discussed in our mini-review. In addition, some articles were surveys that took place during the first wave and are therefore likely not generalizable beyond that.

There are general limitations of many studies that focus on a small and sometimes heterogeneous sample of respondents. Many articles that supposedly focused on MDs did not discuss the specific impact on each MD as experienced by patients; rather, they focus on the delivery of services, acceptability, training, and guidelines. In fact, little is known about the impact of the online setting on the treatment of first-episode psychosis (49), eating disorders (54) or developmental disorders (50). An exception was found in a work on trauma therapy, which reported a barrier to teletherapy for PTSD patients with cognitive impairment (44). Notably, the presence of parents is necessary for children with developmental disorders (50), which might be considered a regression in the psychoanalytical therapy process, which, for instance, sometimes helps a parent and child to separate from each other.

Patients receiving therapy online while staying at home might express their satisfaction, saying that their anxiety about going out is reduced and they feel more in control. These aspects were reported as beneficial, but they could be considered drawbacks as well; avoiding going outside does not do much for the treatment of social anxiety disorders, and being more in control could result in a reinforcement of defense mechanisms or an increase in OCD symptoms.

This mini-review was limited to a Pubmed search and should be considered as a preliminary step towards a systematic review of the literature including other keywords and databases. From this point of view, it would be interesting if the diversity of psychotherapists and the laws that govern their practice around the world were considered. Indeed, online practice has challenged state boundaries more than ever before, which is a positive development, but raises many clinical, legal (55, 56) and ethical questions that are crucial to the profession.

## 5. Conclusion

This review of the literature reveals important qualitative feedback from therapists of diverse horizons regarding how online sessions have become embedded in therapy practice. The use of teletherapy does not seem to be transient, nor does it depend on the COVID-19 pandemic. It appears that acceptability is relatively good from the point of view of both patients and therapists, and it seems that we are witnessing a generalization of hybrid practices in mental health. A possible problem with telepsychotherapy and teleconsultations is how they will be used by ministries of health at the global level. It is feared that online consultations will be imposed for supposedly cost-saving reasons. Not all conditions are suitable for teleconsultation, and in some cases, therapists and patients want to see each other in person. In other words, we fear a loss of therapeutic effects if telepsychotherapy becomes “the new normal”; however, hybrid practice is desired by most therapists and patients. Future studies should examine the effectiveness of the different therapy modalities by comparing three groups of patients: one receiving in-person therapy only, one receiving telepsychotherapy only, and one receiving hybrid services. Finally, the rise of telepsychotherapy might be summed up by a paradoxical sentence that is of great significance in the history of clinical practice: keeping in touch (37) while losing touch (57).

## Author contributions

All authors listed have made a substantial, direct, and intellectual contribution to the work and approved it for publication.

## Funding

This study was supported by JSPS Kakenhi (grant number 19 K12975).

## Conflict of interest

The authors declare that the research was conducted in the absence of any commercial or financial relationships that could be construed as a potential conflict of interest.

## Publisher’s note

All claims expressed in this article are solely those of the authors and do not necessarily represent those of their affiliated organizations, or those of the publisher, the editors and the reviewers. Any product that may be evaluated in this article, or claim that may be made by its manufacturer, is not guaranteed or endorsed by the publisher.
